# Supplementary material for: Examining the Association Between Food Insecurity, Food Literacy, and Food Intake Among Low-Income Adults in Jeddah, Saudi Arabia: A Cross-Sectional Study
Source: Foods. 2025 Sep 1;14(17):3078. doi: 10.3390/foods14173078 (PMC12428145; doi:10.3390/foods14173078)
Supplement: Supplementary file 1 [file foods-14-03078-s001.zip › foods-3780180-supplementary.pdf]

Table S1. Association Between Demographics and Food Literacy.

	**Food literacy**		
	**Poor** (n = 41)	**Adequate** (n = 442)	***P*-value**
**Gender**			
Female	29 (6.7%)	402 (93.3%)	**<0.001**
Male	12 (23.1%)	40 (76.9%)	
**Age (years)**			
18-24	6 (31.6%)	13 (68.4%)	**<0.001**
25-34	8 (13.6%)	51 (86.4%)	
35-44	11 (6.2%)	167 (93.8%)	
45-54	9 (5.4%)	157 (94.6%)	
Above 55	7 (11.5%)	54 (88.5%)	
**Level of education**			
Primary school	11 (7.6%)	134 (92.4%)	0.914
Secondary school	10 (10.3%)	87 (89.7%)	
High school	15 (9.4%)	144 (90.6%)	
Diploma	0 (0%)	19 (100%)	
Bachelor	4 (6.9%)	54 (93.1%)	
Postgraduate education	1 (20%)	4 (80%)	
**Marital status**			
Married	11 (8.9%)	112 (91.1%)	**<0.001**
Single	10 (32.3%)	21 (67.7%)	
Widowed	15 (6.4%)	218 (93.6%)	
Divorced	5 (5.2%)	91 (94.8%)	
**Employment status**			
Full-time employee	1 (2.9%)	34 (97.1%)	0.059
Unemployed	36 (8.4%)	392 (91.6%)	
Part-time employee	4 (22.2%)	14 (77.8%)	
Self-employed	0 (0%)	2 (100%)	

**Number of children**			
No children	11 (21.6%)	40 (78.4%)	0.069
One child	0 (0%)	34 (100%)	
2-3 Children	12 (7.4%)	151 (92.6%)	
4-5 Children	10 (7.8%)	118 (92.2%)	
> 5 Children	8 (7.5%)	99 (92.5%)	
**Household size**			
1-2 people	2 (6.3%)	30 (93.8%)	0.496
3-4 people	12 (6.9%)	163 (93.1%)	
5 people or more	27 (9.8%)	249 (90.2%)	
**Housing structure**			
Lone person	2 (28.6%)	5 (71.4%)	0.369
Couple without children	0 (0%)	1 (100%)	
Couple with child/children	7 (8.2%)	78 (91.8%)	
Father with child/children	1 (10%)	9 (90%)	
Mother with child/children	15 (6.2%)	227 (93.8%)	
Extended family	15 (11.2%)	119 (88.8%)	
Compound family	1 (25%)	3 (75%)	
**Family income, SR/month**			
2000- Less than 3000	19 (7.5%)	236 (92.5%)	0.354
3000- Less than 4000	10 (7.3%)	127 (92.7%)	
4000- Less than 5000	9 (13.6%)	57 (86.4%)	
5000 and above	3 (12%)	22 (88%)	
**Living situation**			
Homeowner	5 (6.8%)	69 (93.2%)	0.053
Renter	27 (7.6%)	330 (92.4%)	
Resident of assisted living facility/residential care accommodation	9 (17.3%)	43 (82.7%)	
**Meal preparation and frequency**			
Prepare no meals	14 (37.8%)	23 (62.2%)	**<0.001**
Prepare some meals	6 (4.8%)	120 (95.2%)	
Prepare most meals	6 (4%)	143 (96%)	

Prepare all meals	15 (8.8%)	156 (91.2%)

Data are *n* (*n*%). Data are analyzed using Chi-square test or Fisher's exact test, as appropriate。Significant *p*-values are shown in bold.

Table S2. Association Between Demographics and Food Insecurity Levels

	**Food insecurity**				
	**Food secure** (*n* = 100)	**Mild food insecurity** (*n* = 85)	**Moderate food insecurity** (*n* = 120)	**Severe food insecurity** (*n* = 169)	***P*-value**
**Gender**					
Female	86 (20.3%)	77 (18.2%)	108 (25.5%)	152 (35.9%)	0.703
Male	14 (27.5%)	8 (15.7%)	12 (23.5%)	17 (33.3%)	
**Age (years)**					
18-24	4 (21.1%)	4 (21.1%)	1 (5.3%)	10 (52.6%)	**0.014**
25-34	10 (17.2%)	10 (17.2%)	13 (22.4%)	25 (43.1%)	
35-44	33 (19%)	21 (12.1%)	56 (32.2%)	64 (36.8%)	
45-54	35 (21.3%)	40 (24.4%)	42 (25.6%)	47 (28.7%)	
Above 55	18 (30.5%)	10 (16.9%)	8 (13.6%)	23 (39%)	
**Level of education**					
Primary school	33 (23.1%)	26 (18.2%)	28 (19.6%)	56 (39.2%)	**0.014**
Secondary school	10 (10.5%)	14 (14.7%)	34 (35.8%)	37 (38.9%)	
High school	30 (19.2%)	32 (20.5%)	37 (23.7%)	57 (36.5%)	
Diploma	3 (15.8%)	3 (15.8%)	8 (42.1%)	5 (26.3%)	
Bachelor	22 (39.3%)	10 (17.9%)	13 (23.2%)	11 (19.6%)	
Postgraduate education	2 (40%)	0 (0%)	0 (0%)	3 (60%)	
**Marital status**					
Married	23 (19%)	12 (9.9%)	40 (33.1%)	46 (38%)	**0.012**
Single	11 (35.5%)	7 (22.6%)	2 (6.5%)	11 (35.5%)	

Widowed	49 (21.6%)	50 (22%)	57 (25.1%)	71 (31.3%)	
Divorced	17 (17.9%)	16 (16.8%)	21 (22.1%)	41 (43.2%)	
**Employment status**					
Full-time employee	12 (35.3%)	5 (14.7%)	7 (20.6%)	10 (29.4%)	0.059
Unemployed	82 (19.5%)	73 (17.4%)	109 (26%)	156(37.1%)	
Part-time employee	4 (22.2%)	7 (38.9%)	4 (22.2%)	3 (16.7%)	
Self-employed	2 (100%)	0 (0%)	0 (0%)	0 (0%)	
**Number of children**					
No children	19 (38.8%)	11 (22.4%)	4 (8.2%)	15 (30.6%)	**0.031**
One child	6 (17.6%)	6 (17.6%)	11 (32.4%)	11 (32.4%)	
2-3 Children	30 (18.9%)	29 (18.2%)	48 (30.2%)	52 (32.7%)	
4-5 Children	29 (23%)	19 (15.1%)	34 (27%)	44 (34.9%)	
> 5 Children	16 (15.1%)	20 (18.9%)	23 (21.7%)	47 (44.3%)	
**Household size**					
1-2 people	4 (12.5%)	5 (15.6%)	12 (37.5%)	11 (34.4%)	0.327
3-4 people	43 (25.6%)	32 (19%)	43 (25.6%)	50 (29.8%)	
5 people or more	53 (19.3%)	48 (17.5%)	65 (23.7%)	108(39.4%)	
**Housing structure**					
Lone person	2 (33.3%)	1 (16.7%)	0 (0%)	3 (50%)	0.182
Couple without children	0 (0%)	0 (0%)	1 (100%)	0 (0%)	
Couple with child/children	18 (21.4%)	8 (9.5%)	27 (32.1%)	31 (36.9%)	
Father with child/children	1 (10%)	1 (10%)	3 (30%)	5 (50%)	
Mother with child/children	47 (19.8%)	45 (19%)	63 (26.6%)	82 (34.6%)	
Extended family	32 (24.2%)	30 (22.7%)	25 (18.9%)	45 (34.1%)	
Compound family	0 (0%)	0 (0%)	1 (25%)	3 (75%)	
**Family income, SR/month**					
2000- Less than 3000	35 (14%)	43 (17.2%)	61 (24.4%)	111(44.4%)	**<0.001**
3000- Less than 4000	29 (21.5%)	24 (17.8%)	42 (31.1%)	40 (29.6%)	
4000- Less than 5000	23 (35.4%)	12 (18.5%)	13 (20%)	17 (26.2%)	
5000 and above	13 (54.2%)	6 (25%)	4 (16.7%)	1 (4.2%)	
**Living situation**					
Homeowner	22 (29.7%)	17 (23%)	18 (24.3%)	17 (23%)	0.181

Renter	68 (19.5%)	60 (17.2%)	90 (25.9%)	130(37.4%)	
Resident of assisted living facility/residential care accommodation	10 (19.2%)	8 (15.4%)	12 (23.1%)	22 (42.3%)	
**Meal preparation and frequency**					
Prepare no meals	6 (16.2%)	6 (16.2%)	8 (21.6%)	17 (45.9%)	**0.02**
Prepare some meals	22 (17.6%)	17 (13.6%)	28 (22.4%)	58 (46.4%)	
Prepare most meals	34 (23.4%)	32 (22.1%)	38 (26.2%)	41 (28.3%)	
Prepare all meals	38 (22.8%)	30 (18%)	46 (27.5%)	53 (31.7%)	

Data are *n* (*n*%). Data are analyzed using Chi-square test or Fisher's exact test, as appropriate. Significant *p*-values are shown in bold.
